# DNA Barcode Goes Two-Dimensions: DNA QR Code Web Server

**DOI:** 10.1371/journal.pone.0035146

**Published:** 2012-05-04

**Authors:** Chang Liu, Linchun Shi, Xiaolan Xu, Huan Li, Hang Xing, Dong Liang, Kun Jiang, Xiaohui Pang, Jingyuan Song, Shilin Chen

**Affiliations:** 1 Institute of Medicinal Plant Development, Chinese Academy of Medical Sciences, Peking Union Medical College, Beijing, People’s Republic of China; 2 School of Computer Science and Engineering, Beijing University of Aeronautics, Beijing, People’s Republic of China; 3 Pidit Inc, Edison, New Jersey, United States of America; American Museum of Natural History, United States of America

## Abstract

The DNA barcoding technology uses a standard region of DNA sequence for species identification and discovery. At present, “DNA barcode” actually refers to DNA sequences, which are not amenable to information storage, recognition, and retrieval. Our aim is to identify the best symbology that can represent DNA barcode sequences in practical applications. A comprehensive set of sequences for five DNA barcode markers ITS2, *rbcL*, *matK*, *psbA-trnH*, and *CO1* was used as the test data. Fifty-three different types of one-dimensional and ten two-dimensional barcode symbologies were compared based on different criteria, such as coding capacity, compression efficiency, and error detection ability. The quick response (QR) code was found to have the largest coding capacity and relatively high compression ratio. To facilitate the further usage of QR code-based DNA barcodes, a web server was developed and is accessible at http://qrfordna.dnsalias.org. The web server allows users to retrieve the QR code for a species of interests, convert a DNA sequence to and from a QR code, and perform species identification based on local and global sequence similarities. In summary, the first comprehensive evaluation of various barcode symbologies has been carried out. The QR code has been found to be the most appropriate symbology for DNA barcode sequences. A web server has also been constructed to allow biologists to utilize QR codes in practical DNA barcoding applications.

## Introduction

The DNA barcoding technology uses a short standard piece of DNA sequence for species identification and has gained wide acceptance as a standard and effective method for biodiversity research, conservation genetics, wildlife forensics, and so on. The 648 bp region of the mitochondrial cytochrome *c* oxidase subunit I (*CO1*) gene has been accepted as the DNA barcode for animals [1], [2]. For plants, two chloroplast genes, namely, *rbcL* and *matK*, were proposed by the plant working group of the Consortium for Barcode of Life (http://www.barcodeoflife.org/) as core barcodes [3] after integrating the results obtained from a number of studies [4], [5], [6], [7], [8], [9], [10], [11], [12]. More recently, the intergenic transcribed spacer (ITS) and its subsequence (ITS2) have also been proposed as additional core barcodes [13]. Furthermore, *psbA-trnH* remains as a supplementary DNA barcode for further evaluation [14]. For fungi, ITS was proposed as the core barcode in the fourth International Barcode of Life Conference (Adelaide, Australia 2011). In summary, through numerous studies, consensus has been reached for core barcodes for animals and plants to date.

With the determination of the core DNA barcodes for the two kingdoms of life, efforts would now start shifting to practical applications of DNA barcoding technologies. At present, “DNA barcode” actually refers to DNA sequences, which has several limitations in practical applications. First, it lacks information compression, which results in a large printout size. Second, it encounters difficulty in information retrieval through direct scanning of DNA sequences. Consequently, adopting a new format to represent DNA barcode sequences is urgently needed to display and retrieve DNA barcode information efficiently.

Barcode technology has been adopted in the manufacturing and retailing industries for many years. Thus, investigating if these well-developed technologies can be applied to represent the so-called DNA barcode would be logical. Actually, a study suggested the use of PDF417 symbology for the “DNA Barcode” [15], which affords efficient information retrieval. However, no comprehensive evaluations of the available barcode types for suitability in encoding DNA barcode sequences have been reported to date. Furthermore, no computational tools have been developed that allow users from a wide range of research communities, industries, and regulatory agencies to utilize barcode symbologies for DNA barcoding applications.

In the current study, a systematic comparison of various one-dimensional (1D) and two-dimensional (2D) barcoding symbologies have been conducted using the sequences of the five most widely accepted plant and animal barcodes (ITS2, *rbcL*, *matK*, *psbA-trnH*, and *CO1*) as test data. Quick response (QR) code was identified as the most appropriate symbology to represent DNA barcodes. A web server was then developed that allow users to utilize QR codes in practical DNA barcoding applications.

## Results

### 1. Comparison of 1D and 2D Barcode Types

The original barcodes are 1D and have been widely used in commercial products, electronic tickets, and so on. Two-dimensional barcodes are developed later and offer several advantages. A comparison of 1D and 2D barcodes based on several major characteristics are shown in Table S1. Then, 53 types of 1D and 10 types of 2D barcodes (listed in Table S2) were selected to test their abilities and characteristics when coding DNA barcode sequences.

**Figure 1:**
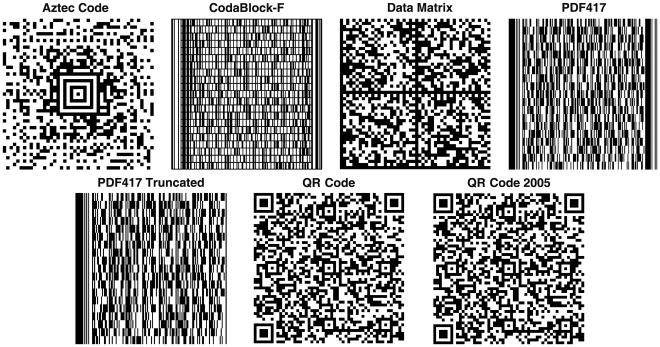
Examples of the seven different types of 2D barcodes used in the current study. An ITS2 sequence from *P. ginseng* (GenBank accession: HQ112416.1) was used as the input.

#### 1.1. Correlation of 2D barcode sizes and the lengths of input DNA barcode sequences

**Figure 2:**
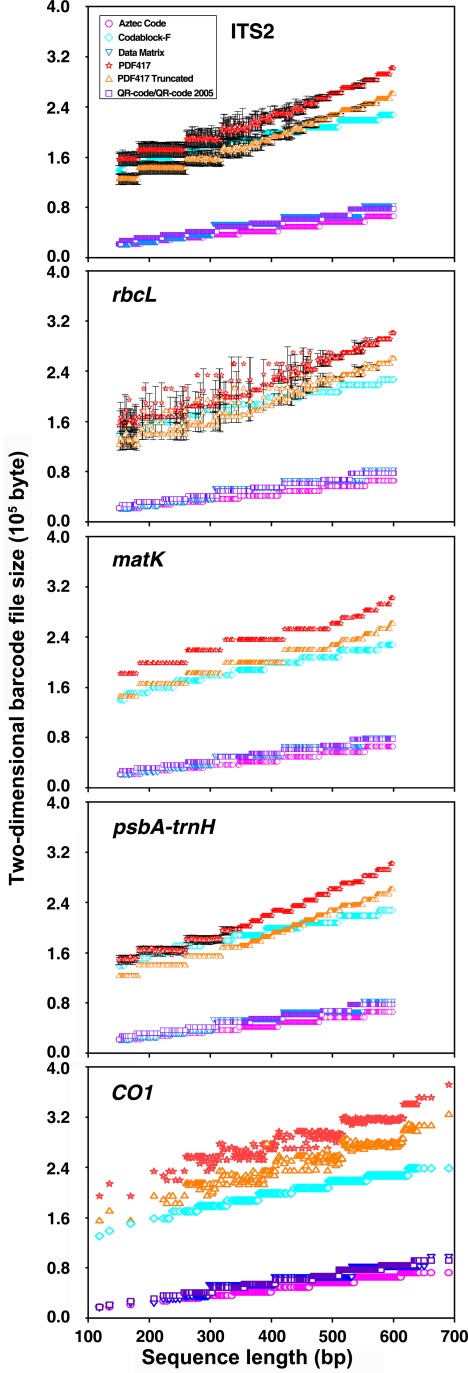
Correlation of image file sizes of seven 2D barcode types and sequence length for five DNA barcode markers.

Sequences from the five most popular plant and animal barcodes, namely, ITS2, *rbcL*, *matK*, *psbA-trnH*, and *CO1*, were used as our test data. The 1D barcode commonly used to describe DNA barcodes was unable to encode even the sequences of ITS2, the shortest DNA barcode with an average length of around 200 bp. Thus, 1D barcode is not practical for encoding DNA barcode sequences. We then went ahead to test the 2D barcodes, the Aztec Code, CodaBlock-F, Data Matrix, PDF417, PDF417 Truncated, QR2005 code, and QR code successfully encoded the sequences from the five DNA barcode sequences. Examples of these seven 2D barcodes are shown using a DNA sequence from *Panax ginseng* ITS2 (GenBank accession: HQ112416) (Fig. 1). Although the theoretical capacities of the 2D barcodes are known (Table 1), they have not been tested with real DNA barcode sequences. We applied the seven 2D barcoding methods to our test data set. It is found that the sizes of the barcodes increase with the length of the DNA barcode sequences derived from the five DNA barcode markers (Fig. 2 and Tables S3). In addition, the barcode sizes of Aztec, Data Matrix, QR and QR2005 are significantly smaller than those of Codablock-F, PDF417, and PDF417 Truncated at various sizes of the input sequence length.

**Table 1:** Comparisons of the characteristics of the seven different types of 2D barcodes.

Name		Aztec Code	CodaBlock-F	Data Matrix	PDF417	PDF417 Truncated	QR code	QR code-2005
Code type		Matrix	Stacked	Matrix	Stacked	Stacked	Matrix	Matrix
Symbol size		15×15 to 27×27 modules	2 to 44 rows	8×8 to 144×144 modules	3 to 90 rows	3 to 90 rows	21×21−177×177 modules	21×21−177×177 modules
Capacity	8-bit bytes	3832	5450	3116	2710	2710	7089	7089
	Numeric	3067	−	2355	1850	1850	4296	4296
	Alphanumeric	1914	2725	1556	1108	1108	2953	2953
Error correction		25%–50%	−	15%∼25%by fixed size	Level 0 to 8	Level 0 to 8	4 steps of 7%	4 steps of 7%

#### 1.2. Compression ratio

**Figure 3:**
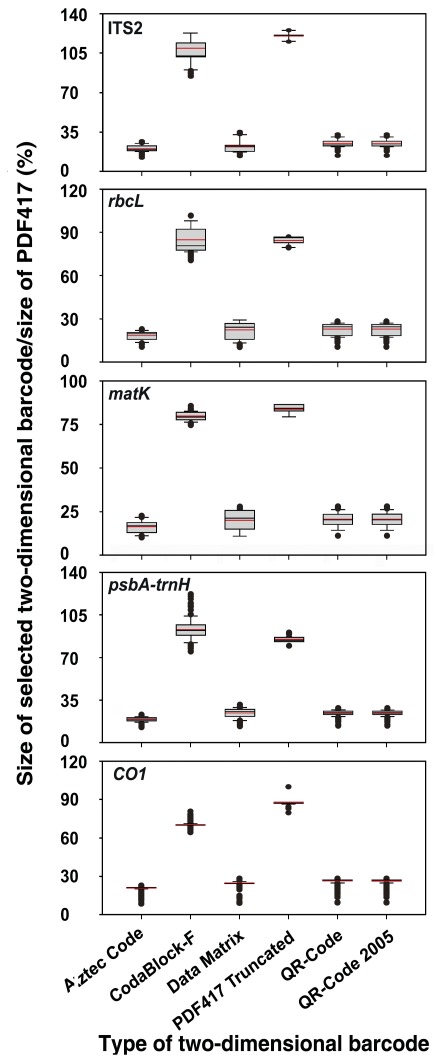
Sizes of six types of 2D barcodes shown as percentage of that of PDF417.

The sizes of the Aztec, CodaBlock-F, Data Matrix, PDF417 Truncated, QR2005, and QR barcodes were normalized to that of PDF417 for the same DNA sequence to calculate the compression ratio of each barcode. As shown in Figure 3, similar to what are shown in Figure 2, Aztec Code, Data Matrix, QR code, and QR code 2005 have the smallest image size among the seven barcodes (Fig. 3). In particular, QR code and QR code 2005 are of the same size. The size ratios of Aztec Code, Data Matrix, and QR code to PDF417 are 16.99%–20.95%, 20.49%–24.73%, and 20.75%–26.47%, respectively, with the average sizes of the three methods being 18.02%, 23.06%, and 23.15%, respectively (Fig. 3).

### 2. Development of a Web Server Supporting QR Code-based DNA Barcoding

**Figure 4:**
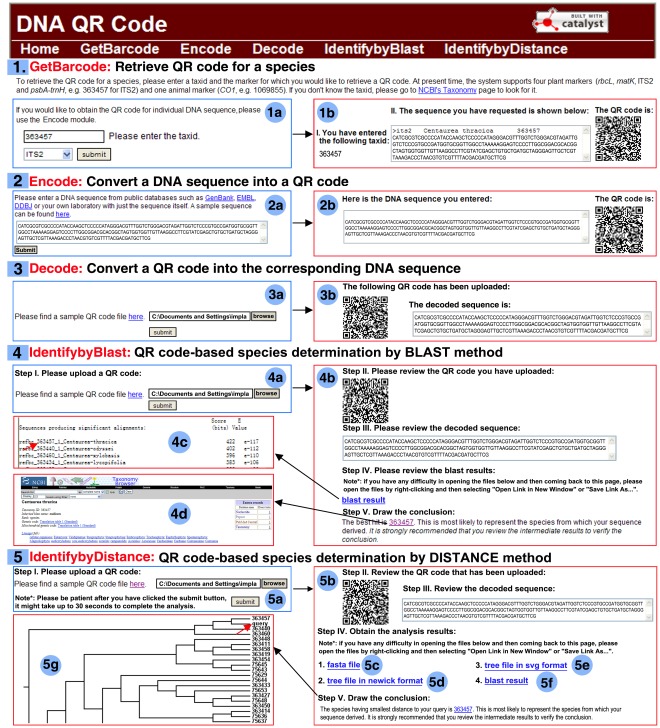
Screenshots of the QRforDNA web server. The module numbers are shaded in blue squares. The module names are shown in Red. The front and the results pages are framed in blue and red respectively. Various components on the front page, final result page and intermediate result pages are shaded in blue circles. (1) Module “GetBarcode”; (1a) Front page of the “Retrieve QR code for a species” module; (1b) Result page of the module. (2) Module “Encode”; (2a) Front page for “Convert a DNA sequence into a QR code” page; (2b) Result page showing the generated 2D barcode. (3) Module “Decode”; (3a) Front page for the “Decode a QR code into a sequence” module; (3b) Result page showing the original DNA sequence decoded from an input QR code. (4) Module “IdentifybyBlast”; (4a) Front page for the “Identify by BLAST” module; (4b) Result page for the module; (4c) the actual BLAST search result; and (4d) the best hit from the BLAST result. This is the predicted species identity for the given sample. (5) Module “IdentifybyDistance”; (5a) Front page for the “Identify by distance” module; (5b) Result page for the module; (5c) the fasta file showing the hits among the top 100 best hits and having *E* value <1e-5 from the BLAST search (details described in the text); (5d) the tree file in newick format; (5e) the tree file in svg format; (5f) the BLAST result; and (5g) the closest species found in the phylogenetic tree. This is the predicted identity of the query.

To promote the practical usage of the QR code in DNA barcoding studies and applications, a web server (QRforDNA, freely accessible at http://qrfordna.dnsalias.org) was developed. The web server contains five modules (Fig. 4) whose functions are described below.

#### 2.1. Retrieval of the QR code

Module 1 (Fig. 4-1) allows users to retrieve the sequences of the five barcode regions (ITS2, *rbcL*, *matK*, *psbA-trnH*, and *CO1*) for their species of interest. One barcode is desired to be given for each species. However, a species can have multiple sequences available. Therefore, a consensus sequence for each species was constructed using all available sequences for each marker to represent the species of interest. The input is a taxid from GenBank and is a DNA barcode marker type (Fig. 4-1a). This module returns the QR code for the consensus sequence corresponding to the species (Fig. 4-1b). This GenBank’s taxonomy system is not replicated in our system, and the user can find the taxid and more detailed information about the species and the sequences from the GenBank’s taxonomy browser web site (http://www.ncbi.nlm.nih.gov/taxonomy).

#### 2.2. Conversion between DNA sequence and QR code

Module 2 (Fig. 4-2) acts as a QR encoder, which takes a DNA sequence as input (Fig. 4-2a) and simply returns the QR code (Fig. 4-2b). Module 3 (Fig. 4-3) serves as a QR decoder, which takes a QR code as input (Fig. 4-3a) and decodes it into the original DNA sequence (Fig. 4-3b). These modules can be used when users want to encode and decode any DNA barcode sequences they have.

#### 2.3. QR code-based species identification

Modules 4 and 5 integrate together the QR decoding and species determination steps (Fig. 4-4 and 4-5). The BLAST- and Distance-based methods are implemented for species determination [16]. Both modules take a QR code as input (Fig. 4-4a, Fig. 4-5a). Module 4 performs the species determination task by searching the DNA barcode database using BLAST and presents the identification result (Fig. 4-4b). The BLAST results can be viewed (Fig. 4-4c). The taxid (Fig. 4-4d) of the top BLAST hit is assigned to the query QR code. In contrast, Module 5 determines the species identity using the Distance-based method and present the identification result (Fig. 4-5b). The query sequence is first used to search against the backend reference sequence database. The hits that belong to the top 100 and have an *E* value <1e-5 are retrieved (Fig. 4-5c). The query sequence and these top hit sequences are then subjected to multiple sequence alignment and phylogenetic tree construction. The resulting tree is presented in nwk (Fig. 4-5d) and svg format (Fig. 4-5e). The BLAST result can also be viewed (Fig. 4-5f). The query is assigned to the identity of its closest neighbor on the tree, whose taxid can be viewed in GenBank (Fig. 4-5g).

#### 2.4. An example usage of the web server

Hereafter, we describe a scenario that demonstrates a application using QR codes to identify samples and track them afterward using the DNA barcoding technology. First, collected biological samples are subjected to DNA extraction and sequencing to obtain the DNA barcode sequence. Relevant data regarding the samples, such as collection site, time, DNA sequence, and species identity, among others, are stored in a central database. The DNA sequences are then converted to QR codes, printed out, and used to label the biological samples. Later, a scanner is used to scan the QR codes one at a time, which are then sent to a central server for decoding and database querying. The results are returned to the scanner for displaying. In this way, the goal of efficient sample (genetic) identification and tracking is achieved.

## Discussion

Although barcode technologies have been well developed and various types of barcodes have been widely used, no comprehensive evaluation on their suitability to encode DNA barcodes using real sequences have been performed. The aim of the current study is to identify the best type of barcode to represent DNA barcode sequences. Fifty-three 1D and 10 2D barcodes have been compared using DNA barcode sequences from five DNA core-barcode markers. Based on coding capacity and compression ratio, the frequently used 1D code was found to have no practical use in encoding DNA barcode sequences due to their small capacity. Among several types of 2D barcodes, the QR symbology is the most suitable. The QR code can encode 7,089 numeric and 4,296 alphanumeric characters and 2,953 bytes of binary (8 bits) data [17]. Its compression efficiency on real DNA barcode sequences is among the best (Figs. 2 and 3). The QR code has several other superior characteristics [18]. It can be divided into multiple data areas, and information stored in multiple QR code symbols can be reconstructed as a single data symbol, allowing high error tolerance. The QR code can also be easily scanned, and algorithms to decode it are well developed. Adopting the QR code as the standard DNA barcode representation format will advance the practical applications of DNA barcodes.

The current study focuses on the representation of DNA barcodes. DNA sequences can also be compressed before they are converted to QR codes, allow the storage of even longer DNA sequences, or reduce further the display size of DNA barcodes. Several DNA sequence compression algorithms have also been developed. These include those encoded DNA sequence into binary strings using various entropy coding methods–from fixed codes, such as the Golomb [19] and Elias [20] codes, to variable codes, such as the Huffman codes [21]–and those that employ statistical pattern matching, such as palindromes, string comparisons, repeat detection, data permutation, and so on [22], [23], [24], [25], [26]. Furthermore, because QR codes can encode different types of data, combining DNA barcode sequences and other types of metadata such as taxonomic information and etc, in a standard format before converting them to QR codes is also possible.

The potential uses of DNA barcoding technologies have been extensively illustrated, such as in the determination of endangered species to prevent smuggling, determination of invasive species for quarantine, determination of species in medicinal herbs to ensure product safety, and so on. One of the most symbolic visions of DNA barcoding is the ultimate creation of a handheld DNA barcoder that contains components for automatic DNA extraction, DNA amplification, DNA sequencing, and a DNA barcode analysis engine that incorporates the associated software tools and databases. Such “Life Barcoder” will not only be used to identify species but will also be linked via the World Wide Web to other kinds of biodiversity data, such as images and related information about that species. However, the realization of the “Life Barcoder” requires a standard format to represent the DNA barcode sequences. The current study conducted a comprehensive comparison of various barcode types and found that the QR code can be used to represent DNA barcode sequence efficiently. The results and tools obtained from this study would promote DNA barcoding applications to a more practical level.

## Materials and Methods

### 1. Data Set Used in the Current Study

The sequences for four plant DNA barcodes, namely, ITS2, *rbcL*, *matK* and *psbA-trnH* were parsed from GenBank record files using custom Perl scripts. And *CO1* sequences were retrieved from GenBank (Version 188) by searching GenBank with the query “barcode”[keyword]. The numbers of *CO1* sequences retrieved from GenBank were significantly larger than those of the other markers. Encoding all *CO1* sequences into various types of barcode symbologies becomes prohibitively expensive in computation. As a result, only the *CO1* sequences for Lepidoptera (butterflies and moths) were used in the analysis comparing the barcode symbologies, as this set of data are representative of all *CO1* sequences in terms of length distribution and sequence composition. However, the entire set of *CO1* sequences was included in the backend reference databases of our web server. The downloaded sequences were checked for orientations. After the removal of the flanking sequences, only those having a length between 150 and 600 bp for the four plant DNA barcodes and between 100 and 700 bp for the *CO1* barcodes were kept for compression-ratio analysis. The sequences used in the comparison are included in Files S1, S2, S3, S4, S5. The sequences used in the web server will be updated regularly. It should be pointed out, although many taxonomy systems are available, the GenBank’s system was used because it is the only system that links the taxonomy ids (taxid) to DNA sequences.

### 2. Comparison of Various Barcode Types

We used Barcode Studio (TEC-IT, Austria, version 7.5) to encode each sequence of the test dataset into each of the 53 1D and 10 2D symbologies. A custom Perl script was used to summarize the percentage of DNA barcode sequences that can be encoded with each symbology. For those symbologies that can potentially encode DNA barcode sequences, the ratios between the sizes of these barcodes to that of PDF417 for the same sequences were calculated. PDF417 was used as the reference because it was proposed to represent DNA barcode previously [15].

### 3. Implementation of the QR Code-based Species Identification Module

We only implemented the two most basic methods for species determination in the web server at present time. These two methods, the BLAST and the Distance-based method, are based on local and global sequence similarities [16]. Additional species determination methods can be added to the web server in the future. Regardless which method is used, the QR-code is first decoded into the corresponding DNA sequence and the individual-level DNA barcode sequence database is used for analysis. For the BLAST method, the query sequence was used to search the database using BLAST. All significant hits (*E* value <1e-40) were retained and the species identity of the top hits was assigned to the query sequence. For the Distance-based method, a pre-filtering step is added because the tree construction process with the query and all sequences in the database is very time-consuming. The query sequence is first used to search against the backend sequence database. The hits that rank within the top 100 and have an *E* value <1e-5 are retrieved. The query sequence and these retrieved sequences are then subjected to multiple sequence alignment and phylogenetic tree construction using neighbor joining algorithm and P distance metric implemented in Clustalw (version 1.82) with default parameters. The query is assigned to the identity of its closest neighbor on the tree. Because of the well-known problems of the presence of insertions and inversions in the *psbA-trnH* sequences, these sequences are subjected to additional pre-processing steps using custom scripts, including the identification and removal of the rps19 insertions, and the identification and reverse-complementation of the inversions.

### 4. Implementation of the Web Server

To construct species-level consensus sequence, as well as individual-level reference DNA barcode sequence database, all sequences for the four plant barcodes (ITS2, *rbcL*, *matK*, and *psbA-trnH*) and the animal barcode (*CO1*) from GenBank (Version 188) were downloaded and processed. The QR encoding and decoding algorithms were implemented using JAVA language. The QRforDNA web server application was developed with the Perl Catalyst Framework (5.80024) using MySQL (5.1.44) as the backend database management system. The web server was deployed on an Apache server (2.2.14) running on a Fedora 12 Linux operating system and can be freely accessed at http://qrfordna.dnsalias.org. We have tested the web server on the window platform using Internet Explorer (version 6.0, 7.0 and 8.0) and Firefox (version 9.0 and 10.0), and the Mac platform using Firefox (version 10.0) and Safari (version 5.1.2).

## Supporting Information

Table S1 Comparisons of 1D and 2D symbologies.
(DOC)

Table S2 Types of 1D and 2D barcodes tested in the present study.
(DOC)

Tables S3 **Table S3-Table S7 are presented in the associated Excel file because of the large size of the tables.**
(RAR)

Table S3 Sizes for 2D barcodes encoded from DNA sequences for ITS2.
(XLS)

Table S4 Sizes for 2D barcodes encoded from DNA sequences for *rbcL*.
(XLS)

Table S5 Sizes for 2D barcodes encoded from DNA sequences for *matK*.
(XLS)

Table S6 Sizes for 2D barcodes encoded from DNA sequences for *psbA-trnH*.
(XLS)

Table S7 Sizes for 2D barcodes encoded from DNA sequences for *CO1*.
(XLS)

File S1 Fasta sequences for ITS2 marker used in the current study.
(RAR)

File S2 Fasta sequences for *rbcL* marker used in the current study.
(RAR)

File S3 Fasta sequences for *matK* marker used in the current study.
(RAR)

File S4 Fasta sequences for *psbA-trnH* marker used in the current study.
(RAR)

File S5 Fasta sequences for *CO1* marker used in the current study.
(RAR)
