# Supplementary material for: DNA Barcode Goes Two-Dimensions: DNA QR Code Web Server
Source: PLoS One. 2012 May 4;7(5):e35146. doi: 10.1371/journal.pone.0035146 (PMC3344831; doi:10.1371/journal.pone.0035146)
Supplement: Table S1 — Comparisons of 1D and 2D symbologies. (DOC) [file pone.0035146.s001.doc]

**Table S1.** Comparisons of 1D and 2D symbologies

| **Characteristics** | **1D barcode** | **2D barcode** |
| --- | --- | --- |
| Data format | Alphabet, number, and symbol | Alphabet, number, symbol, photograph, voice, finger print, electronic signature |
| Data storage capacity | About 20 bytes | About 2,000 bytes |
| Data density | Low | High |
| Symbol size | Symbol length increases with data amount (misreading is possible) | Square (minimization possible) |
| Reading speed | Fast | Influenced by the amount of data |
| Reading direction | Reads only in one direction | Reads in 360° |
| Error detection and fault-correction function | Possible error detection but restoration is impossible | Possible error detection and restoration |
| Electronic digital signature encryption | Not available and not required | Available and required |
