# Supplementary material for: DNA Barcode Goes Two-Dimensions: DNA QR Code Web Server
Source: PLoS One. 2012 May 4;7(5):e35146. doi: 10.1371/journal.pone.0035146 (PMC3344831; doi:10.1371/journal.pone.0035146)
Supplement: Table S2 — Types of 1D and 2D barcodes tested in the present study. (DOC) [file pone.0035146.s002.doc]

**Table S2.** Types of 1D and 2D barcodes tested in the present study

| Type | Name of Barcode |
| --- | --- |
| 1D barcode | [Codabar](http://en.wikipedia.org/wiki/Codabar) 2 widths, [Code 11](http://en.wikipedia.org/wiki/Code_11), [Code 128](http://en.wikipedia.org/wiki/Code_128), [Code 128 SubsetA](http://en.wikipedia.org/wiki/Code_128A), [Code 128 SubsetB](http://en.wikipedia.org/wiki/Code_128A), [Code 128 SubsetC](http://en.wikipedia.org/wiki/Code_128A), Code 2 of 5 DataLogic, Code 2 of 5 IATA, Code 2 of 5 Industry, Code 2 of 5 Interleaved, Code 2 of 5 Matrix, Code 2 of 5 Standard, [Code 32](http://en.wikipedia.org/wiki/Code_39), [Code 39](http://en.wikipedia.org/wiki/Code_39), Code 39 Full ASCII, [Code 93](http://en.wikipedia.org/wiki/Code_93), Code 93 Full ASCII, EAN 13, EAN 13 + 2 Digits, EAN 13 + 5 Digits, EAN 14 (GTIN 14), EAN 8, EAN 8 + 2 Digits, EAN 8 + 5 Digits, EAN/UCC 128, Flattermarken, ISBN 13, ISBN 13 + 5 Digits, ISMN, ISSN, ISSN + 2 Digits, ITF 14 (GTIN 14), LOGMARS, MSI, NVE 18, Pharmacode One-Track, Pharmacode Two-Track, Plessey, Plessey Bidirectional, PZN7, PZN8, SSCC 18, Telepen, Telepen Alpha, UCC 128, UPC 12, UPC A, UPC A + 2 Digits, UPC A + 5 Digits, UPC E, UPC E + 2 Digits, UPC E + 5 Digits, VIN / FIN |
| 2D barcode | 2D-code, Aztec Code, Codablock-F, Data Matrix, MaxiCode, Micro PDF417, Micro QR-Code, PDF417, PDF417 Truncated, QR-Code, QR-Code 2005, |
